# The Use of Ipecacuanha Spray in Winter Cough and Bronchitic Asthma

**Published:** 1874-11

**Authors:** Sidney Ringer, William Murrell


### MISCELLANEOUS.

### On Ipecacuanha Spray in Winter Cough and Bronchitic Asthma.

By SIDNEY RINGER, M. D.; and WILLIAM MURRELL.

The successful use of a secret remedy by a well-known practitioner induced us to try the effect of inhalation of ipecacuanha spray. Our results have been so satisfactory that we desire to draw the attention of the profession to this mode of treating these obstinate complaints — winter cough and bronchial asthma. Our observations were made during January and February. Whilst under this treatment the patients took only colored water, and continued their usual mode of living in all respects.

We shall first refer to winter cough. We have made observations on twenty-five patients, whose ages varied between forty-five and seventy-two, with one exception—that of a woman of thirty-two years. We purposely chose severe cases. In order to avoid burdening this paper with too much detail, we give here a typical case, which will serve in most points to illustrate the condition of the patients. Subsequently we shall report two actual cases in full.

The patient has been troubled with winter cough perhaps for many years. During the summer he is pretty well; but during the cold months, from October to May, he suffers sometimes without intermission, occasionally getting a little better and then catching cold; or perhaps he may lose his cough for a few weeks, but again takes cold on the slightest exposure. So short is the breathing that he can walk only a few yards, especially in the cold air, and finds it hard work to get up stairs, and is often quite unfitted for active life. The breathing grows worse at night, so that he cannot sleep unless the head is propped up with several pillows. He is troubled, too, with paroxysmal dyspnœa, usually at night, which may last several hours, and compels him to sit up. Sometimes the breathing is difficult only on exertion; and in those cases it is made much worse by fogs, east winds, or damp. The expectoration varies greatly; in a few cases there is very little; usually, however, it is rather abundant, and consists of mucus or pus, often with little or no rhonchus in the chest. It is often difficult to expel the expectoration. The cough is generally very violent, frequent, hacking and paroxysmal, and the fits may last ten or twenty minntes, and even excite vomiting. They are generally brought on by exertion; nay, in bad cases so easily are they provoked that the patient is afraid to move, or even to speak. The cough and expectoration are much worse in the morning on waking. Sometimes the cough is slight, and then the expectoration is generally scanty, the distressed breathing being the chief symptom. The patient generally wheezes badly, especially at night, and in a bad case the legs are swollen. The patient is emphysematous; there is often no rhonchus, or only sonorous aud sibilant or a little bubbling rhonchus at both bases.

In this common but obstinate complaint our results have been very striking, although in many of our patients so bad was the breathing that, on being shown into the out-patients' room they dropped into a chair, and for a minute or so were unable to speak, or only in monosylables, having no breath for a long sentence. We used the ordinary spray producer, with ipecacuanha wine pure or variously diluted. On the first application it sometimes excites a paroxysm of coughing, which generally soon subsides, but if it continues a weaker solution should be used. The patient soon becomes accustomed to it, and inhales the spray freely into the lungs. At first a patient inhales less adroitly than he learns to do afterwards, as he is apt to arch his tongue so that it touches the soft palate, and consequently less enters the chest than when the tongue is depressed. The spray may produce dryness or roughness of the throat, with a raw sore sensation beneath the sternum, and sometimes it causes hoarseness; whilst, on the contrary, some hoarse patients recover voice with the first inhalation. As they go on with the inhalation, they feel it getting lower and lower into the chest till many say they can feel it as low as the ensiform cartilage

The dyspnœa is the first symptom relieved. The night after the first application the paroxysmal dyspnœa was often improved, and the patient had a good nights rest, although for months before the sleep was much broken by shortness of breath and coughing. The difficulty of breathing on exertion is also quickly relieved; for often after the first admiuistration the patient walked home much easier than he came to the hospital, and this improvement is continuous, so that in one or two days or a week the patient can walk with very little distress, a marked improvement taking place immediately after each inhalation; and although after some hours the breathing may again grow a little worse, yet some permanent improvement is gained, unless the patient catches a fresh cold. We have heard patients say that in a weeks time they could walk two miles with less distress of breathing than they could walk a hundred yards before the spray was employed. In some instances two or three days daily spraying is required before any noticeable improvement takes place, this comparatively slow effect being sometimes due to awkward inhalation, so that but little ipecacuanha passes into the bronchial tubes. The effect on the cough and expectoration is also very marked. these both greatly decrease in a few days, though the improvement in these respects is rather slower than in the case of the breathing. Sometimes for tne first few days the expectoration is rather increased. It speedily alters in character, so that it is expelled much more readily, and thus the cough becomes easier, even before the expectoration diminishes.

Treated in this way the patient is soon enabled to lie down at night with his head lower, and in a week or ten days, and sometimes earlier can do with one pillow. This improvement occurs in spite of fogs, damp or east winds--even,while the weather gets daily worse, and when the patient is exposed to it the chief part of the day. All these patients came daily to the hospital. Of course it is much better to keep the patient in a warm room.

Here are short notes of two cases, the first a very successful one:

J. H——, aged seventy-two, has had a winter cough for the last three years. The cough comes on in fits, and is very bad at night. Fogs greatly aggravate it. She spits about a teacupful of thick yellow phlegm in the twenty-four hours. So bad is her breath that she cannot lie down at night, but is propped up with pillows, and is always wheezing. She is obliged to stay at home for weeks together. Her lungs are emphysematous, with only sonorous râles. After the first inhalation there was great improvement—freedom from cough all night, with much easier breathing. Further improvement took place after the next days inhalation, and still more after the third, so that on the sixth day of treatment, and after three inhalations she reported that her breathing "was not near so troublesome; thinks nothing of it now; does not spit up half as much," and the expectoration is white and frothy. This poor woman was loud in her praise of the treatment; said "she never expected it," and "when first she came to the hospital thought she should never get about again." J. H—— is now sufficiently recovered to take charge of a shop, though before her attendance at hospital she had not been out of her room for four months. She was discharged, and called a month afterwards to say there had been no relapse.

Now comes a less tractable instance, a fair specimen of one of the more obstinate cases.

M. A——, aged thirty-two, came to the hospital January 29th with a wintercough of many years standing, and worse this winter than ever before. The cough is paroxysmal, the slightest exertion, even talking, bringing on an attack. The paroxysms vary much, but generally last ten minutes. In the twenty-four hours she spits about a teacupful of thick yellow phlegm. Extremely short-breathed, and she is quite unable to do her housework, and at night is unable to sleep unless propped up with three pillows and a bolster. The breathing always gets worse at night. Fogs increase all her troubles. Has been hoarse for weeks, and if she talks much she altogether loses her voice. Her chest is very sore with coughing, She is emphysematous, and her breath-sounds are obscured by cooing râles.

Feb. 3d.--The patient, who has had an inhalation on five successive days, now says she is in every way much better. The breathing is much easier; the cough is not nearly so violent; her chest is less sore; the expectoration is much less; and there is very little hoarseness.

6th.—The inhalations have been continued daily. The patient says she is better than she has been all the winter. The improvement in her breathing is very great, and she can now do with only one pillow at night instead of three. She sleeps much better. The cough is greatly improved, and instead of being "aggravated" towards night, is now better at that time. Expectoration has almost ceased.

10tn.—Has had only one inhalation since last date, and her breathing has been a little more distressed.

12th.—Has had an inhalation daily, and the dyspnœa has again nearly disappeared.

17th.—Has had but one inhalation since last date. The cough now has almost left her, and she often goes twelve hours without a fit. Her breathing is so much better that she now does her own house-work, and is not propped up at night.

Discharged after ten inhalations and nineteen days treatment.

A month afterwards she came to the hospital to say that her breathing was all right, and that she had been perfectly well since her discharge, with the exception of a slight hacking cough.

All but one of the twenty-five patients were benefitted. In one case the improvement was very gradual, but there was evident temporary improvement after each inhalation. In twenty-one cases the average number of inhalations required was 9·4, and the average number of days was twelve, before the patients were discharged cured. The greatest number of inhalations in one case was eighteen, and the smallest three. The case longest under treatment required twenty-four days; the shortest, four.

In employing the ipecacuanha spray, in order to ensure as far as possible only its topical effects, we were careful to direct the patient to spit out and even to rinse out the mouth at each pause in the administration, for a much larger quantity of the wine collects in the mouth than passes into the lungs. If this precaution is not adopted, sometimes enough is swollowed to excite nausea and even vomiting, by which means the bronchial mucus is mechanically displaced, and of course in this way effects temporary improvement. Even when this precaution was observed, a protracted inhalation will excite nausea and sometimes vomiting by the absorption of the wine by the bronchial mucous membrane; though, strange to say, when thus induced, vomiting was long delayed, even for several hours — nay, sometimes not till the evening, though the inhalation was used in the morning. In the reported cases, however, improvement was not due to the nauseating effects of the spray, for we took care to avoid this contingency by administering a quantity inadequate to produce this result. The duration of each inhalation will depend on the amount of spray produced by each compression of the elastic ball, and on the susceptibility of the patient to the action of ipecacuanha. As a rule, the patient at first will bear from twenty squeezes of the spray without nausea, and will soon bear much more. After two or three squeezes, especially on the commencement of the treatment, we must pause a while. It is necessary to look at the patient's tongue and tell him to learn to depress it, for if the tongue is much arched it will hinder the passage of the spray to the lungs. It is a good plan to tell the patient to close his nose with his fingers and to breathe deeply. The inhalation should be used at first daily; afterwards every other day suffices, and the interval may be gradually extended. If the ipecacuanha wine is diluted, then the spray must be used a longer time. In cold weather the wine should be warmed.

We have tried the spray with very satisfactory results in a few cases of the following more severe though closely allied disease:— A patient for several years has suffered from severe winter cough, with much dyspnœa, cough, and expectoration; and on several occasions has spat up a considerable quantity of blood. The physical signs denote slight fibroid consolidation, with excavation of both apices, and much amphysema, perhaps atrophous in kind. There is little or no rhonchus, and no fever. The expectoration may be slight or very abundant, muco-purulent or purulent. The dyspnœa is, perhaps, very severe; and is so paroxysmal as to justify calling the case bronchial asthma, with emphysema, and fibroid phtisis. In these cases the ipecacuanha spray is almost as beneficial as in the preceding. It soon controls the dyspnœa, thus enabling the patient to sleep, and greatly lessens expectoration and cough; and by these means really improves the general health. As in the previous cases, the first inhalation may considerably improve the breathing, though the effects are not so permanent, the dyspnœa returning in the evening; so that spraying is needed night and morning, and may be necessary for weeks or months, the ipecacuanha appearing rather to give relief than to permanently cure the dyspnœa.

We have used the spray in two cases of true and severe bronchial asthma, with very opposite results. In one severe case. accompanied by a great deal of bronchitis, it gave very great relief. The other patient, not so ill, has been all his life asthmatical; and on catching even a slight cold his breathing becomes greatly oppressed. In this instance each application of the spray considerably aggravated the dyspnœa, even when the wine was diluted with an equal quantity of water. Possibly a still weaker solution might have been borne; but we are inclined to think that in this case any quantity of ipecacuanha would have disagreed, as the tightness of breathing increased almost immediately the inhalation was begun.

The successful case was a very severe one. For years this woman had suffered from bronchitic asthma, and when she applied to the hospital was unable to lie down owing to shortness of breath. She suffered also from violent paroxysmal dyspnœa, the worst attack beginning about three o'clock, A. M., compelling her to start out of bed and struggle for breath. She was very emphysematous; her voice was very hoarse. The first inhalation removed the hoarseness, and much improved her breathing, which continued freer till midnight, when the dyspnœa returned. The cough was eased, and she expectorated more freely. Each inhalation always gave her very great and marked relief. She walked to the hospital with great difficulty, and was constrained to stop frequently. On entering the room she could not speak, but labored violently and with loud wheezing to get her breath. A few inhalations would gradually set the breathing free, so that the air entered more and more, and the wheezing gradually left, till, on the completiou of the inhalation, she could breathe without difficulty. As the breathing improved she could feel the spray descend lower and lower in her chest. At first it would seem to reach only the back of the tongue, then the top of the sternum, then descend to midsternum, and at last she felt as if it reached as low as the pit of the stomach. This improvement was maintained through the day, but at evening a relapse would occur, so that her nights, though at first bad, were still better than before the treatment. Soon, however, the effects became more lasting, and she slept well. On discontinuing the spray, however, her breathing again grew worse, and she was obliged to revert to the treatment; but unfortunately she so soon caught cold, and so bad was the weather, that she was obliged to stay away for days together. Whilst her breathing improved the cough and expectoration also improved, but these two symptoms continued rather troublesome. Probably in bad bronchitic asthma the spray must at first be used twice a day or oftener, and must be continued for some time to ward off dyspnœa, for in these obstinate chronic cases the bronchitis may take a considerable time to cure. So marked was the improvement from the spray that the patient and her friends expressed their astonishment, especially at the prompt relief it gave.—*London Lancet.*

